# Exploratory crossover field study indicates efficacy and feasibility of light therapy glasses to mitigate fatigue in multiple sclerosis

**DOI:** 10.1038/s41598-026-56950-3

**Published:** 2026-06-23

**Authors:** Julia Ottersbach, Markus Canazei, Sophie K. Bauer, Celine Hfalek, Caren Berggold, Thomas C. Wetter, De-Hyung Lee, Ralf A. Linker, Roland F. J. Popp

**Affiliations:** 1 Department of Psychiatry and Psychotherapy, Center of Sleep Medicine, University of Regensburg, 93053 Regensburg, Germany; 2 Department of Experimental Psychology, University of Regensburg, 93053 Regensburg, Germany; 3 Department of Psychology, University of Innsbruck, 6020 Innsbruck, Austria; 4 Department of Neurology, University of Regensburg, 93053 Regensburg, Germany

**Keywords:** Fatigue, Multiple sclerosis, Light therapy, Blue-enriched bright light, Diseases, Health care, Medical research, Neurology, Neuroscience

## Abstract

Fatigue is highly prevalent, clinically significant, and persistent in multiple sclerosis (MS), highlighting the need for effective countermeasures. To evaluate the feasibility and efficacy of blue-enriched bright light (BL) therapy glasses in reducing MS-related fatigue, this single-blind, randomized, active-controlled crossover study included 20 patients with MS with significant fatigue (Fatigue Severity Scale [FSS] total score > 36). Participants used blue light (BL) glasses or dim red light (DRL) glasses for 20 min daily after waking. Each intervention lasted 1 week with a 6-day washout between conditions. Fatigue, measured using the FSS four times per intervention week, was the main exploratory outcome measure. Further, the acute overall effects of both interventions were assessed using a Visual Analogue Scale for Fatigue (VAS_F) immediately before and after each intervention and at 13:00. Quality of life (Multicultural Quality of Life Index) was rated four times per week. Feasibility was evaluated using self-reported comfort, side effects, and sleep quality. Both interventions significantly decreased total FSS scores on the sixth and seventh intervention days (follow-up), with BL inducing clinically significant reductions on the sixth day. Both interventions significantly reduced VAS_F ratings immediately after the intervention and at 13:00, with BL showing significantly more reduction. No effects on quality of life were observed. Patients reported greater comfort, improved well-being, and reduced fatigue with the BL glasses. Side effects were low and subjective sleep quality was unaffected. Despite its small sample size, the present exploratory study provides initial evidence for light therapy glasses as a feasible, immediately effective intervention for MS-related fatigue.

**Supplementary Information:**

The online version contains supplementary material available at 10.1038/s41598-026-56950-3.

## Introduction

Fatigue, characterized by disproportionate tiredness and exhaustion, is one of the earliest, most prevalent, and most disabling symptoms in MS, affecting up to 85% of patients within the first year and rising to 95% with disease progression^1–4^. The precise mechanisms underlying MS-related fatigue remain elusive, with studies discussing structural damage to white and grey matter, inflammatory processes, a maladaptive network recruitment, and metacognitive interpretations of brain states linked to helplessness^1^. From a pathogenetic point of view, a distinction can be drawn between primary and secondary fatigue in MS. Primary fatigue is attributed directly to disease-related mechanisms such as disrupted cortico-subcortical connectivity due to lesions, inflammatory processes, and neuroendocrine dysregulation caused by central nervous system lesions. Secondary fatigue arises from secondary factors such as medication side effects, comorbidities, and MS-related symptoms^5,6^. Although the distinction between primary and secondary fatigue is theoretically valuable and may inform diagnostic and therapeutic strategies, differentiating these components in individual patients with MS remains challenging. Most patients exhibit overlapping forms, making clear classification difficult^7,8^. Unlike more objective neurological symptoms, fatigue is inherently subjective and lacks reliable biomarkers, complicating its assessment and contributing to inconsistencies in epidemiological and clinical research^9,10^. Common measurement tools include the Fatigue Severity Scale (FSS^11^), the Modified Fatigue Impact Scale (MFIS^12,13^), and Visual Analogue Scales for Fatigue ranging from 0 to 100. Given the frequent comorbidity of fatigue with sleep disorders, depression, and cognitive impairment, a comprehensive diagnostic approach is recommended^14^. Fatigue can manifest in all stages of MS^15^ irrespective of neurological impairment^12^. It affects social and professional life, severely impairs quality of life and compromises mental health^15–17^. The consequences of this for the everyday life of patients with MS are severe, underscoring the urgent need for effective countermeasures.

Pharmacological treatments, including with amantadine, modafinil, and methylphenidate, have demonstrated no greater efficacy in reducing MS-related fatigue than placebo, and frequently induce adverse effects^18^. Non-pharmacological interventions, encompassing physical or psychological approaches, appear to hold greater promise^19^. These include exercise therapy^20^, aerobic training^21,22^, resistance training^23^, cognitive behavioural therapy^24^, and mindfulness-based methods^25^.

Light therapy is an evidence-based treatment for seasonal affective disorder (SAD), typically involving exposure to polychromatic bright white light between 2,500 and 10,000 lx^26^, In addition to its use in treating affective disorders, research has shown promising results for mitigating fatigue in patients with cancer^27^, Parkinson’s disease^28^, and after traumatic brain injury^29^. There is also some evidence that blue-enriched light therapy with significantly lower illuminance levels (e.g., 750 lx) is effective in treating SAD^30^ or mitigating sleepiness and sustained attentional lapses following sleep deprivation^31^.

The underlying mechanisms of the alerting effects of light include the body’s pacemaker, the suprachiasmatic nucleus. Light, being its strongest synchronizer, induces acute alerting effects both during day and night^32,33^ and affects sleep, mood, alertness, wakefulness, and the stability of the circadian rhythm^34–37^. Light therapy, utilizing these effects, is commonly administered using light boxes, but the use of light therapy glasses, providing light via the glasses’ frames, has recently gained some traction, as this approach can easily be integrated into daily life. Light therapy glasses have been shown to be effective in improving sleepiness and mood in medical inpatients^38^, in treating depression in adolescents^39^, and in reducing fatigue in patients with breast cancer^40^ and patients with (non)Hodgkin lymphoma^41^.

Two studies have examined the effects of light therapy on MS-related fatigue. Mateen et al.^42^ and Voggenberger et al.^43^ both compared the efficacy of bright light therapy (10,000 lx) to that of dim red light (300 lx) administered via light boxes. In the first study, patients were exposed twice daily for 1 h over the course of 4 weeks^42^. In the study of Voggenberger et al.^43^, light therapy was applied once daily for 30 min in the morning (within 3 h of waking up) over the course of 2 weeks. In both studies, exposure to bright and dim light resulted in significant reductions in MS-related fatigue to the same extent.

To the best of our knowledge, no study has examined the effects of blue-enriched light (BL) therapy on MS-related fatigue or utilized light therapy glasses as an intervention in this context. To address this, the present field study employed a randomized, active-controlled crossover study design, exposing patients with MS with significant fatigue to BL and dim red (DRL) light therapy glasses at home. Light therapy was administered daily immediately after waking up for 1 week in each condition. We hypothesized that both interventions would improve MS-related fatigue after 1 week of usage, with stronger acute effects expected for BL than for DRL within hours of exposure. Given that light exposure was conducted in the morning, we expected no effects of the intervention on subjective sleep parameters during the nights following light exposure. As the BL therapy in this study utilized an illuminance level of 1,500 lx, we further expected higher levels of reported side-effects than with DRL. As an exploratory analysis, we assessed comfort ratings for both light interventions as an additional measure of feasibility.

## Methods

### Study design and light interventions

Following a single-blind, active-controlled, crossover design, this study included two light interventions. For the active (BL) intervention, the Luminette® 3 glasses (Lucimed SA, Wavre, Belgium) with an illuminance of 1,500 lx and peak light emission at 468 nm were used for 20 min immediately upon waking up, which is a typical approach that has proven effective in prior research^28,38,39^. The dim red light (DRL) intervention also utilized Luminette® glasses with the same form factor, but with an illuminance of 150 lx and peak light emission at 660 nm, as used in prior studies as an active control or sham condition^28,44,45^. Both glasses switched off automatically after 20 min of exposure. At study inclusion, patients were randomly assigned to one of two study groups: group A first used DRL for 1 week, followed by a 6-day washout period and a second intervention week with BL; group B started with BL following the same pattern. Participants were informed about testing two different light interventions to improve their MS-related fatigue. The study was conducted in accordance with the World Medical Association Declaration of Helsinki and approved by the ethics committee of the University of Regensburg (ID: 23-3307-101, date: 17 April 2023) The study was registered with the German Clinical Trials Register (ID: DRKS00036464, URL: https://drks.de/search/en/trial/DRKS00036464/entails; date: retrospectively registered on 25 March 2025). Participants received 100€ compensation for study participation.

### Procedure and measurements

Recruiting was conducted using flyers that were disseminated to patients with MS at an outpatient clinic in the Department of Neurology (University of Regensburg), patients of neurologists in private practice, and physiotherapists located in the Regensburg, Germany, area. Additionally, the study was promoted online in patient forums and on the study administration platform “Sona” of the University of Regensburg.

#### Study inclusion and screening

The first contact with potential participants took place via telephone and aimed to check inclusion and exclusion criteria as well as current MS-related fatigue levels.

Thereafter, a comprehensive screening took place at the Center of Sleep Medicine (University of Regensburg) or via video conference. Following a description of the study and its interventions in detail, participants provided written informed consent for study participation and completed screening questionnaires (Table 1 and Supplementary Materials Appendix A) via the smartphone-based survey tool ExpiWell (West Lafayette, IN, USA). This survey instrument was also utilized to administer timed questionnaires throughout the 22-day study period. Participants were informed via push notifications in the smartphone tool about oncoming surveys and were sent reminders if there was a delay in receiving questionnaires responses.

**Table 1:** Questionnaires used during the screening.

Assessment	Measurements and measures
General questions	Demographic data, disorders, medication, report of most recently assessed score on the Expanded Disability Status Scale^a^
Sleep disorders	* Berlin Questionnaire Sleep Apnoea*^b^: obstructive sleep apnoea
	* Restless Legs Syndrome diagnostic criteria*^c^: restless legs syndrome
	* Regensburg Insomnia Scale*^d^: psychophysiological insomnia
Daytime sleepiness	* Epworth Sleepiness Scale*^e^: overall daytime sleepiness, sleep propensity
Fatigue	* Fatigue Severity Scale*^f^: fatigue severity
	* Daily Fatigue Impact Scale*^g^: daily impact of fatigue
	* Visual Analogue Scale for Fatigue:* fatigue level
Sleep quality	* Pittsburgh Sleep Quality Index*^h^: overall subjective sleep quality
Chronotype	* Morningness–Eveningness Questionnaire*^i^
Mental health	* Beck Depression Inventory II*^j^*:* depressive symptoms

^a^^46^; ^b^^47^; ^c^^48^; ^d^^49^; ^e^^50^; ^f^^11^; ^g^^51^, adapted; ^h^^52^; ^I^^53^; ^j^^54^.

#### Test weeks

**Fig. 1:**
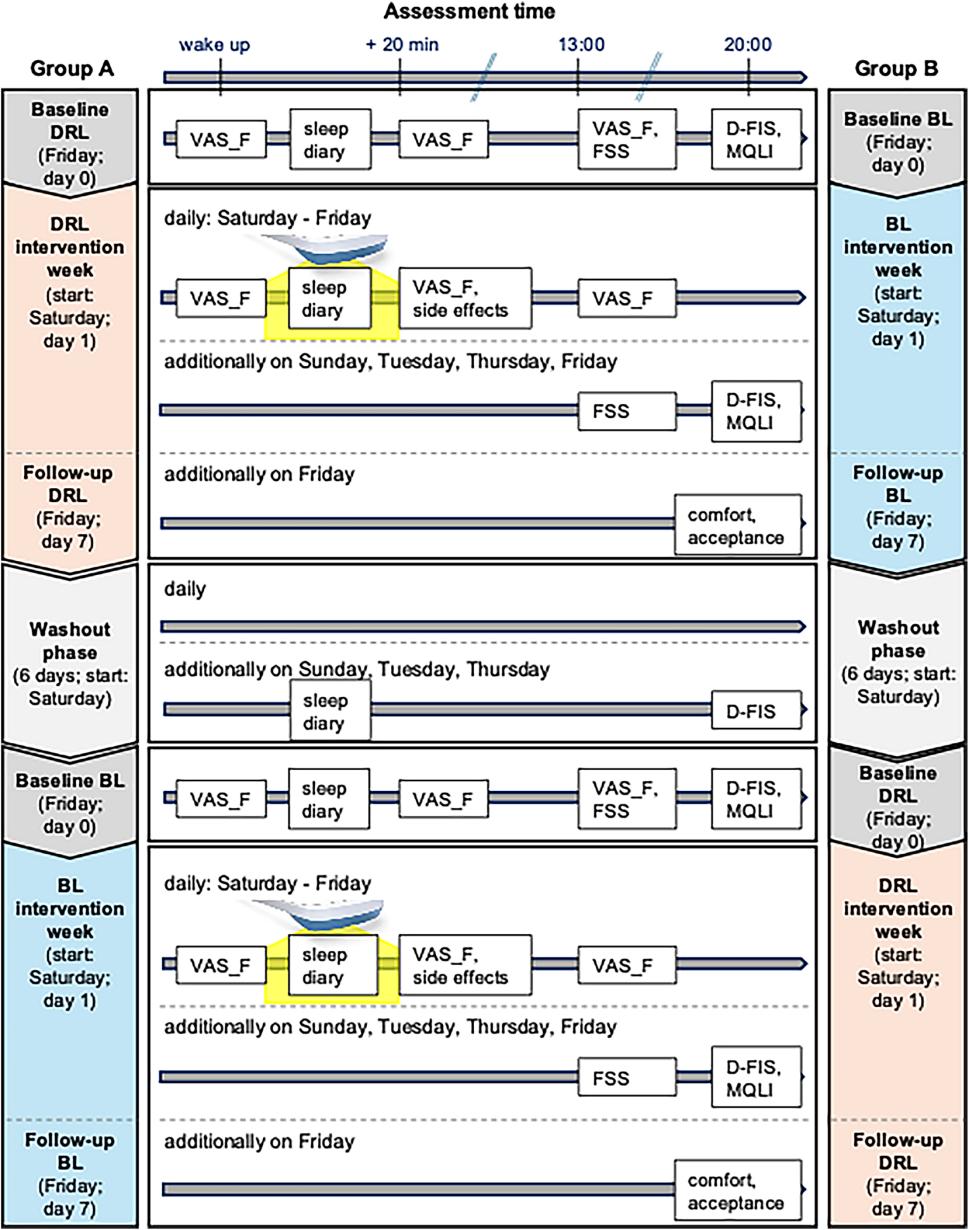
Study flow. *Note*: The yellow bars indicate the daily 20-min light exposure during both intervention weeks starting on saturday and ending on friday; *Abbreviations*: DRL, dim red light; BL, blue enriched bright light; VAS_F, Visual Analogue Scale for Fatigue; FSS, Fatigue Severity Scale; D-FIS, Daily Fatigue Impact Scale; MQLI, Multicultural Quality of Life Index.

Prior to each 7-day intervention period, a baseline assessment was conducted (on Friday; Fig. 1). The Fatigue Severity Scale (FSS) was administered once, the Visual Analogue Scale for Fatigue (VAS_F) was administered three times, and the Daily Fatigue Impact Scale (D-FIS) and Multicultural Quality of Life Index (MQLI) were administered once in each patient.

Each 1-week intervention period started on Saturday (day 1), separated by a 6-day washout period (see Fig. 1). During the intervention periods, participants were required to rate their level of fatigue daily on the VAS_F, immediately after waking up. The light exposure then began, during which participants filled in the sleep diary. The light glasses switched off automatically after 20 min, after which participants rated their fatigue levels on the VAS_F again and filled in the side effects questionnaire. At 13:00, fatigue was again rated on the VAS_F, and on days 2, 4, 6, and 7, participants additionally completed the FSS at 13:00 and the D-FIS and MQLI in the evening (20:00). In addition, participants completed the comfort and acceptance questionnaire once at the conclusion of each intervention period (Friday, follow-up) at 20:00.

During the 6-day washout period, the sleep diary was completed daily, and fatigue was rated every other evening using the D-FIS.

### Study population

#### Inclusion criteria

Consenting participants between 18 and 65 years old, with a medical report of MS diagnosis according to the 2017 McDonald criteria^55^, and with significant fatigue, (FSS total score > 36^11^, were included.

#### Exclusion criteria

The exclusion criteria included pregnant or breastfeeding women and participants with drug or alcohol use disorders, severe neurologic or somatic disorders (such as epilepsy or polyneuropathy), prevailing psychotic symptoms and/or relevant cognitive impairment, impairment in sensory or motor functions that could compromise study participation, an acute MS episode within the past 6 weeks prior to study participation, changes in MS medication within the 6 months prior to study participation, use of more than one psychoactive substance that affects wakefulness (such as amphetamines, sedatives, antidepressants, hypnotics, antihistamines, beta blockers), excessive cigarette consumption (> 15 cigarettes/day); eye diseases (such as retinopathy, retinitis pigmentosa, diabetic retinopathy, macular degeneration, and glaucoma), and intercontinental travel within 6 weeks prior to study participation.

### Outcome measures

Self-reported fatigue, assessed using the FSS total score four times during each intervention period, served as main outcome measure. The VAS_F, ranging from 0 to 100, was administered three times per day to exploratorily and unidimensionally assess the immediate and prolonged effects of light exposure on fatigue levels. The D-FIS, additionally assessing fatigue levels, was completed four times during the intervention period. Further measures included the MQLI four times each intervention week, daily side effects and their severity, and daily sleep logs and comfort ratings at the end of each intervention period, Adherence to the light intervention protocol was assessed by analysing saved logs of the VAS_F questionnaires administered immediately before and after each light treatment.

Table 2 provides a list of all questionnaires and their specific assessment times along with the assessments and outcome measures for the questionnaires utilized. Additional information for each questionnaire can be found in the Supplementary Materials Appendix A.

**Table 2:** Measurements assessed during the two intervention weeks.

Assessment	Measurements and measures	Assessment time
Fatigue	* Fatigue Severity Scale: total score*^a^	At 13:00 on days 2, 4, 6, 7
	* Visual Analogue Scale for Fatigue*: level of fatigue from 0 to 100%	Daily: before and after the intervention and at 13:00
	* Daily Fatigue Impact Scale*^b^	At 20:00 on days 2, 4, 6, 7
Quality of life	* Multicultural Quality of Life Index*^c^	At 20:00 on days 2, 4, 6, 7
Comfort ratings	* Comfort ratings, acceptance*	Last day of the intervention week (day 7, Friday)
Side effects	* Asthenopic complaints*^d^*:* overexertion, headache, watering eyes, itchy eyes, stinging eyes, blurred vision, pain, glare, dizziness	Daily: post intervention
Sleep quality and quantity	* Sleep diary:* sleep quality in %, time in bed, sleep time, wake-up time, naps during the day	Daily: during intervention

^a^^11^; ^b^^51^, adapted; ^c^^56^; ^d^^57^, adapted.

### Data analyses

#### Sample size calculation

We conducted an a-priori power analysis before study start using G*Power v3.1 software^58^ for the Wilcoxon signed-rank test (matched pairs) with an estimated effect size of *d* = .60, as reported by light therapy research for cancer-related fatigue^27^; past research using light therapy for MS-related fatigue has not reported effect sizes^42,43^. With a moderate effect size of *d* = .60, an α error of .05, two-sided testing, and a power of 1 − *β* =  .80, a sample size of 25 participants was deemed sufficient for our within-subject design.

#### Data preparation

All data were examined for missing values and outliers (details in the Supplementary Materials, Appendix B). The original dataset with missing data and the dataset with substituted data (intention-to-treat analysis) were both analysed using parametric and non-parametric statistical tests. All analyses yielded the same significant results. Thus, for FSS, VAS_F, D-FIS, MQLI, and sleep parameters, results from parametric statistical tests (repeated measures analyses of variance; ANOVA) with intention-to-treat analysis are reported.

#### Analyses of carry-over effects

To control for carry-over effects of the light interventions and the efficacy of the washout period, FSS, VAS_F, D-FIS, and MQLI scores from both baseline measurements were compared using the Student’s *t*-test for dependent samples.

#### Comparison of baseline and follow-up scores

Baseline scores of the FSS, VAS_F (immediately after waking up, 20 min later, and at 13:00), D-FIS, and MQLI were compared with values recorded on the last day of the 1-week intervention period (herein called the follow-up) using two-factorial ANOVA with repeated measures with the factors of intervention (DRL, BL) and measurement day (baseline, follow-up).

#### Main outcome measure: fatigue throughout the intervention week

The FSS, administered once (at 13:00 and in the evening, respectively) at baseline and on four intervention days (days 2, 4, 6, and 7) for both interventions, was analysed as main outcome measure to examine the efficacy of the light therapy interventions on patients’ fatigue levels. A two-factor ANOVA with repeated measures was therefore run with the factors of intervention (DRL, BL) and measurement day (baseline and days 2, 4, 6, and 7) for FSS total scores. As an exploratory analysis, FSS scores were analysed inter- and intraindividually regarding the minimally important difference of 4.05, which indicates a clinically significant difference of FSS-measured fatigue^59^.

#### Fatigue throughout the intervention days

Fatigue scores measured with the VAS_F were assessed three times during each intervention day (immediately before and after the intervention and at 13:00) on all seven intervention days (Saturday to Friday) during both interventions (BL, DRL). These were analysed between both conditions to assess the acute effects of the light therapy interventions on fatigue levels immediately after light exposure and hours later. A three-factorial ANOVA with repeated measures including the factors of intervention (DRL, BL), measurement time (immediately before/after the intervention, and at 13:00), and measurement day (day 1 to 7) revealed no significant effect of the factor measurement day. Thus, a two-factorial ANOVA with repeated measures was applied to VAS_F scores for the two factors of intervention (DRL, BL) and measurement time (immediately before/after the intervention and at 13:00).

#### D-FIS-measured fatigue and quality of life throughout the intervention weeks

Data from the D-FIS and MQLI, assessed once (at 13:00 and in the evening, respectively) at baseline and on four intervention days (days 2, 4, 6, and 7) for both interventions, were analysed using two-factor ANOVAs with repeated measures with the factors of intervention (DRL, BL) and measurement day (baseline and days 2, 4, 6, and 7). Results from all analyses regarding the D-FIS can be found in supplementary materials (Appendix C).

#### Sleep parameters, comfort, and side effects ratings

From the daily reported sleep diaries, the parameters of sleep quality (%), total sleep time (min), time in bed (min), sleep latency (min), and sleep efficiency (%) were extracted. Two-factor ANOVAs with repeated measures and the factors of intervention (DRL, BL) and measurement day (baseline to day 7) were run for each sleep parameter to assess effects of the light interventions on nocturnal sleep following light exposure. Furthermore, the total number of naps were calculated for each intervention week and compared using the Student’s *t*-test for dependent measures.

Comfort and side effects ratings were analysed using the Wilcoxon signed-rank test to compare both light interventions in regard to the experienced acceptance and comfort levels as well as side effects occurrence and severity.

#### Statistical testing

For descriptive analyses, mean, standard deviation, standard error, median, and interquartile range were calculated. All graphs show means and standard errors of the mean. For significant ANOVA findings, post-hoc pairwise comparisons were run using Bonferroni-corrected significance levels. In cases of sphericity violations, Greenhouse–Geisser correction was applied to the significance level. All analyses were performed in SPSS software (v29.0, IBM Corp., Armonk, NY, USA) with a significance level of 5% and two-sided testing.

## Results

### Study sample

**Fig. 2:**
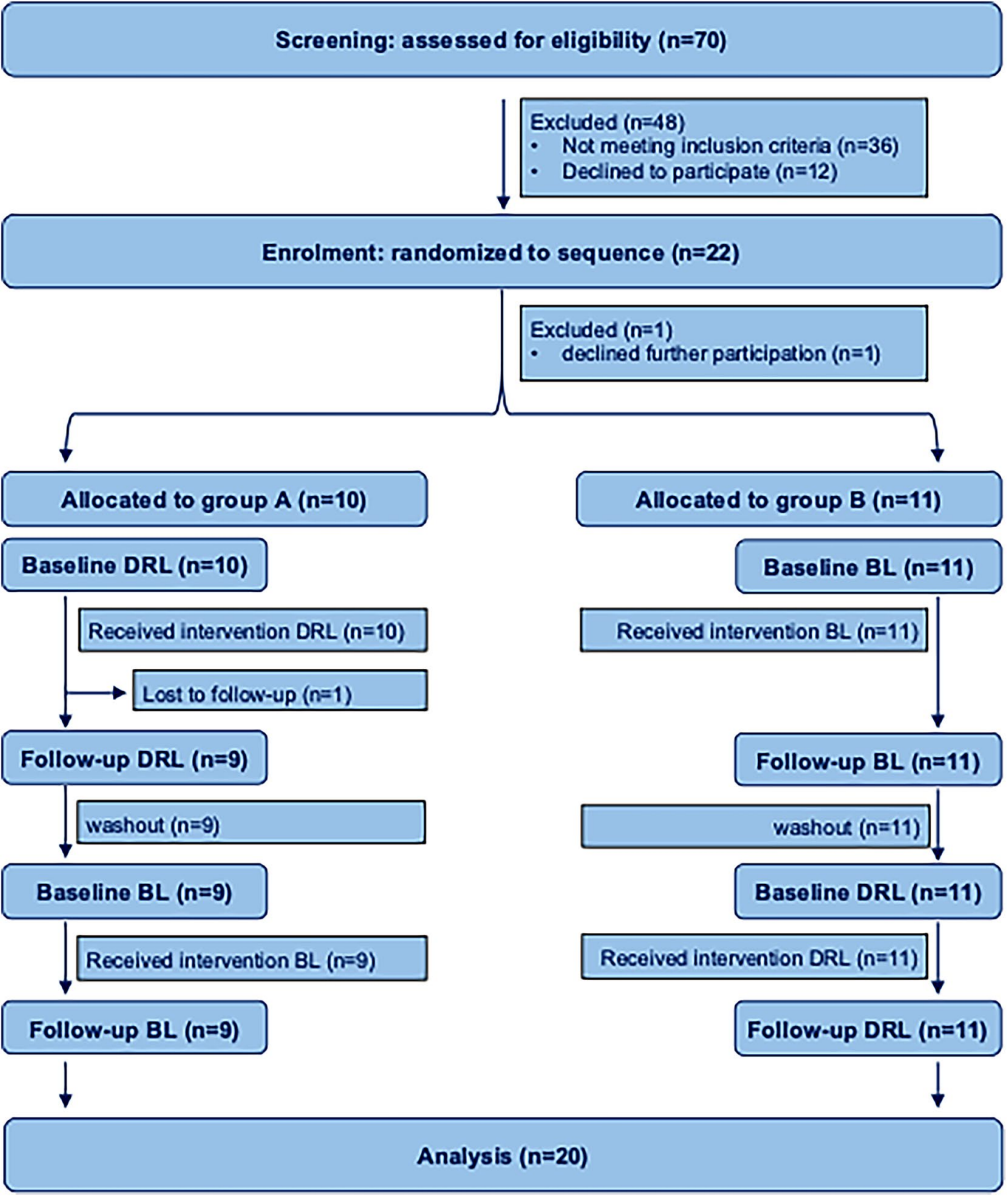
CONSORT 2010 study flow diagram**. ***Note*: n, number; DRL, dim red light intervention; BL, blue-enriched light intervention. Reasons for exclusion were use of psychoactive substance that impairs wakefulness (n = 30), severe smoking (n = 3), severe alcohol intake (n = 1), language barrier (n = 1), and other severe disorders (n = 1).

A total of 70 patients diagnosed with MS were screened between December 2023 and May 2025, of whom 34 were eligible for participation. Most commonly, participants were excluded due to taking more than one psychoactive substance that can affect wakefulness. Of all eligible participants, 12 participants withdrew from the study prior to enrolment, and 1 participant withdrew directly prior to the start of the first intervention week, leaving 21 participants who started the study, of which 20 participants completed it (one participant from group A dropped out during intervention week 1 [DRL condition]). Figure 2 shows the CONSORT study flow diagram.

The final sample consisted of 20 participants aged 23 to 62 years (mean age ± standard deviation 40.5 ± 11.8 years) with 5 men and 15 women (Table 3). Sixteen patients had a diagnosis of relapsing–remitting MS, three were diagnosed with primary progressive MS and one was diagnosed with secondary progressive MS. Disease duration was most often more than 5 years (n = 13). Sixteen patients reported poor levels of sleep (Pittsburgh Sleep Quality Inventory score > 5), 12 participants reported significant levels of insomnia symptoms (measured using Regensburg Insomnia Scale), and 10 participants reported significant levels of depressive symptoms (measured using Beck Depression Inventory-II).

**Table 3:** Demographic and clinical characteristics of the study sample.

Sample size (*n*)	20
Male (*n*)	5
Female (*n*)	15
Age in years (*M* ± *SD [range])*	40.5 ± 11.8 (23–62)
Occupation	
Student (*n*)	4
Working (*n*)	10
Stay-at-home (*n*)	1
Retired (*n*)	5
Diagnosis (McDonald criteria 2017)	
Relapsing–remitting MS (*n*)	16
Primary-progressive MS (*n*)	3
Secondary-progressive MS (*n*)	1
Disease duration in years *(M* ± *SD)*	8.2 ± 7.6
1–3 years (*n*)	4
3–5 years (*n*)	3
More than 5 years (*n*)	13
EDSS score^a^ (*M* ± *SD [range])*	1.9 ± 1.8 [0–6]
MS medication^b^(n)	15
Screening questionnaires	
PSQI (*M* ± *SD [n poor sleep*^c^])	7.8 ± 3.2 (16)
D-MEQ (*M* ± *SD*)	50.4 ± 13.6
Definite evening (*n*)	2
Moderate evening (*n*)	3
Neutral (*n*)	8
Moderate morning (*n*)	6
Definite morning (*n*)	1
OSAS (*n clinically suspicious*^d^)	3
RLS (*n clinically suspicious*^e^)	6
RIS (*M* ± *SD [n clinically suspicious*^f^])	14.6 ± 4.8 (12)
ESS (*M* ± *SD [n clinically suspicious*^g^])	10.6 ± 4.3 (8)
FSS (*M* ± *SD [n clinically suspicious*^h^])	52.3 ± 7.0 (20)
D-FIS (*M* ± *SD*)	19.9 ± 5.0
VAS_F (*M* ± *SD*)	56.5 ± 19.5
BDI-II (*M* ± *SD [n clinically suspicious*^i^])	14.35 ± 8.0 (10)

n, number; *M,* mean, *SD,* standard deviation, MS, multiple sclerosis; EDSS, Expanded Disability Status Scale; PSQI, Pittsburgh Sleep Quality Index; D-MEQ, Morningness–Eveningness Questionnaire German version; OSAS, Berlin questionnaire Sleep Apnoea; RLS, Restless Legs Syndrome; RIS, Regensburg Insomnia Scale; ESS, Epworth Sleepiness Scale; FSS, Fatigue Severity Scale; D-FIS, Daily Fatigue Impact Scale; VAS_F, Visual Analogue Scale for Fatigue; BDI-II, Beck Depression Inventory—II. Revision.

^a^EDSS scores can range from 0 = “normal neurological exam, no disability in any functional system” in steps of 0.5 to 10 = “death due to MS”.

Cut-off values for clinically suspicious scores:

^b^All MS medication was immunomodulatory and included the substances diroximel fumarate (n = 1), ofatumumab (n = 4), ocrelizumab (n = 3), teriflunomide (n = 1), fingolimod (n = 1), natalizumab (n = 1), dimethyl fumarate (n = 2), natalizumab (n = 1), and ozanimod (n = 1). Five patients did not take any MS-specific medication.

^c^PSQI cut-off score > 5.

^d^OSAS cut-off score of ≥ 2 in 2 or more out of 3 categories.

^e^RLS cut-off score = 1 on all RLS criteria.

^f^RIS cut-off sum score > 12.

^g^ESS cut-off sum score > 10.

^h^FSS cut-off sum score > 36.

^i^BDI-II cut-off sum score > 14.

### Adherence and control for carry-over effects

#### Adherence to the study protocol

Adherence to the study protocol was indirectly monitored using the saved time stamps of the VAS_F questionnaires recorded before and after each light treatment. Of the maximum number of 280 interventions for the complete sample, 8 VAS_F ratings immediately before the start of the light exposure and 13 VAS_F ratings immediately after light exposure were missing, resulting in an adherence rate of 96.3%. Furthermore, the mean time difference between the two VAS_F ratings was 33 ± 13 min.

#### Carry-over effects

To control whether the washout period was effective to prevent carry-over effects of the first intervention week, baseline scores from both intervention weeks were compared using the Student’s *t*-test for dependent variables. Patients’ reported fatigue levels (VAS_F, FSS) and quality of life (MQLI) did not differ between the two baseline measurements (all *p* > .05, Table S1, Supplementary Materials Appendix C), demonstrating an effective wash-out.

### Efficacy

#### Comparison of baseline and follow-up scores

A two-factor repeated measures ANOVA revealed a significant main effect of measurement day (F[1, 19] = 13.418, *p* = .002, partial η^2^ =  .414), with significantly lower FSS scores at follow-up (46.2 ± 7.8) than at baseline (49.5 ± 8.1, *p* = .002), but no main effect for intervention and no interaction effect were seen (all *p* >  .05) for the assessed FSS scores (see Table 4).

**Table 4:** Descriptive statistics of baseline and follow-up scores.

	DRL		BL		Total sample	
	Baseline (n = 20)	Follow-up (n = 20)	Baseline (n = 20)	Follow-up (n = 20)	Baseline (n = 40)	Follow-up (n = 40)
	*M* ± *SD*	*M* ± *SD*	*M* ± *SD*	*M* ± *SD*	*M* ± *SD*	*M* ± *SD*
VAS pre	52.3 ± 24.4	48.8 ± 25.8	51.0 ± 25.5	51.5 ± 23.7	51.1 ± 24.6	50.1 ± 24.5
VAS post	43.3 ± 22.8	39.5 ± 22.8	48.8 ± 21.5	38.0 ± 18.7	46.0 ± 22.1	38.8 ± 20.6
VAS 13:00	51.0 ± 26.1	41.5 ± 22.5	59.0 ± 22.0	37.3 ± 20.0	55.0 ± 24.2	39.4 ± 21.1
FSS	48.8 ± 8.1	46.2 ± 8.8	50.1 ± 8.0	46.3 ± 7.0	49.5 ± 8.0	46.2 ± 7.8
MQLI	60.4 ± 17.2	64.4 ± 14.6	65.2 ± 9.4	65.3 ± 15.9	62.8 ± 13.9	64.8 ± 15.1

DRL, dim red light intervention; BL, blue-enriched light intervention; *M*, mean, *SD*, standard deviation, VAS, Visual Analogue Scale for Fatigue, FSS, Fatigue Severity Scale, Daily Fatigue Impact Scale, MQLI, Multicultural Quality of Life Index.

A three-factor ANOVA including the factors of measurement time (immediately before and after the intervention, and at 13:00), measurement day (baseline, follow-up), and intervention (BL, DRL) for all assessed VAS_F scores (Table 4) revealed a significant interaction effect between measurement time and measurement day (F[2, 38] = 7.390, *p* = 0.002, partial η^2^ = 0.280). Post-hoc tests showed significantly lower VAS_F scores at follow-up immediately after the intervention (38.8 ± 20.6) than immediately before the intervention (50.1 ± 24.5 *p* = 0.02), but there were no other significant differences (all *p* > 0.05). Further, we found a significant main effect of measurement day (F[1, 19]  = 7.772, *p* = 0.012, partial η^2^ = 0.290), with significantly lower VAS_F scores at follow-up (42.8 ± 22.5) than at baseline (50.7 ± 23.7, *p* = 0.012). We found no interaction effects between measurement time and condition, measurement day and condition, or between measurement time, measurement day, and condition (all *p* > 0.05). Further, no main effect was found for condition (*p* > 0.05).

Regarding quality of life scores (Table 4), no main effects or interaction effect were found in a two-factor ANOVA with repeated measures (all *p* > 0.05).

#### Fatigue throughout the intervention weeks

**Fig. 3:**
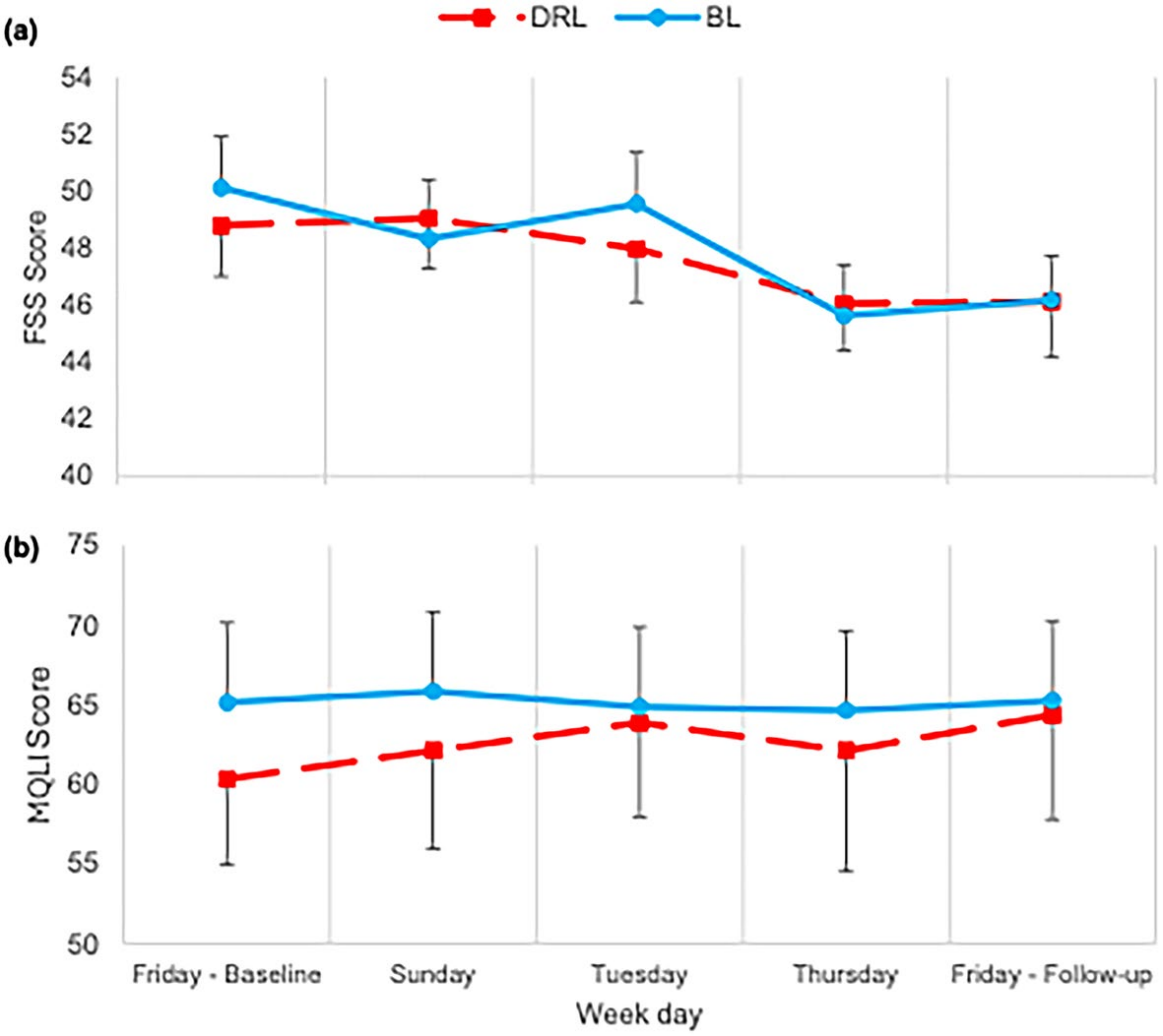
Fatigue and quality of life scores over the course of the week. *Note*: (**a**) Fatigue Severity Scale (FSS); and (**b**) Multicultural Quality of Life Index (MQLI) for the DRL (dim red light, red dashed line) and BL intervention (blue-enriched light, blue line). Error bars indicate the standard error of the mean.

FSS values (Fig. 3a) were included in a two-factorial repeated measures ANOVA with the factors intervention (DRL, BL) and measurement day (baseline, day 2, day 4, day 6, day 7), revealing a significant main effect for measurement day (F[4, 76] = 5.884, *p* < 0.001, partial η^2^ = 0.236), with significantly lower FSS values on day 6 (Thursday, 45.9 ± 7.6, *p* = 0.024) and day 7 (Friday [follow-up], 46.2 ± 7.8, *p* = 0.017) than at baseline (Friday, 49.5 ± 8.0). No main effect for intervention or an interaction effect was found (*p* > 0.05). Regarding the minimally important difference of 4.05 on the FSS total score, which would constitute a clinically significant difference in outcome measures according to Rooney et al.^59^, this was achieved for all patients using BL with a mean reduction in FSS scores from 50.13 at baseline (Friday) to 45.63 on day 6 (Thursday), but no reductions of 4.05 or more were seen on other intervention days. Αt follow-up, the minimally important difference was missed by 0.10, with a mean reduction in FSS scores of 3.95. In the DRL intervention, the group-based FSS baseline scores were not reduced by the minimally important difference on any intervention day.

When examining each patient’s individual changes in FSS total scores from baseline, a clinically significant difference was found in 11 patients when using DRL (4 on day 4 [Tuesday], 7 on day 6 [Thursday], and 8 on day 7 [Friday, follow-up]) and in 15 patients when using BL (6 on day 2 [Sunday], 3 on day 4 [Tuesday], 10 on day 6 [Thursday], and 6 on day 7 [Friday, follow-up]). Of these patients, three showed clinically significant differences only with DRL, and seven showed clinically significant differences only with BL.

#### Fatigue throughout the intervention days

**Fig. 4:**
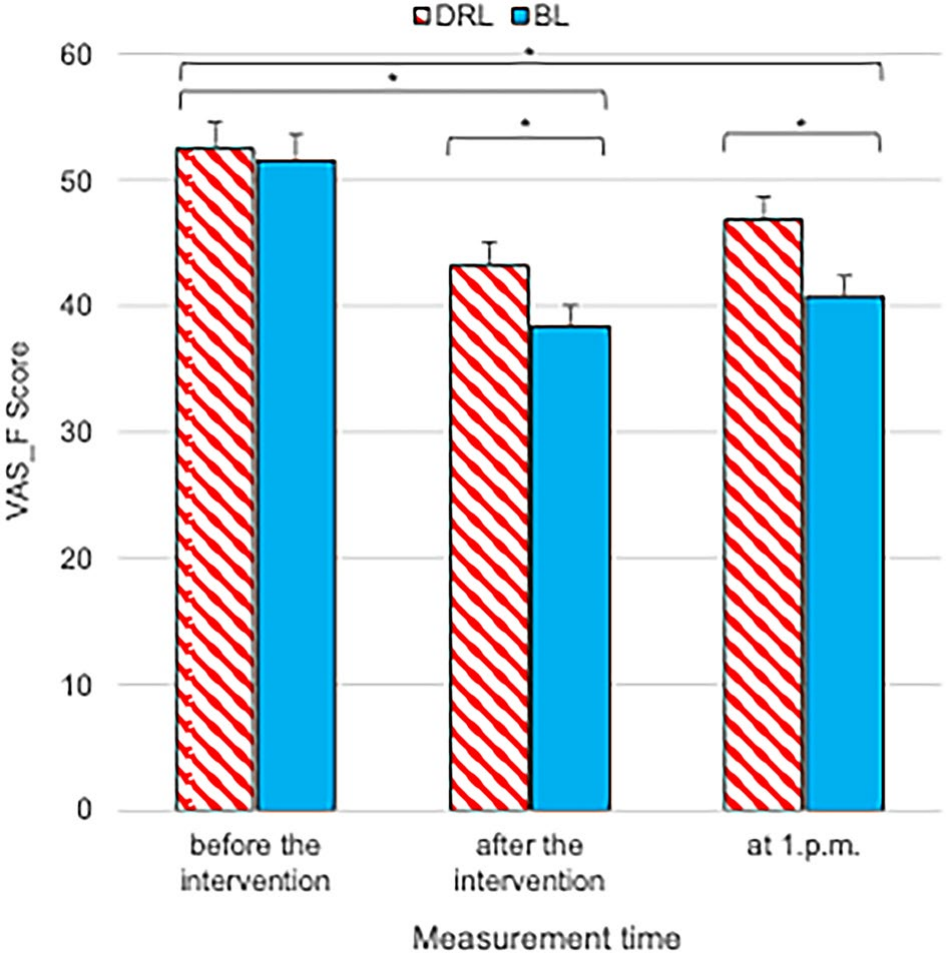
Visual analogue scale for fatigue (VAS_F) scores. *Note*: Included are all measures from intervention days (day 1 until day 7 [follow-up]) in the DRL (dim red light [DRL], red shaded bar) and BL intervention (blue-enriched light, blue bar) immediately before and immediately after the intervention and at 13:00. Error bars indicate the standard error of the mean. *significant at *p* < 0.05.

A two-factor repeated measures ANOVA including the factors intervention (DRL, BL) and measurement time (immediately before and after the intervention, and at 13:00) of patients’ VAS_F scores showed a significant interaction effect between measurement time and intervention (F[1.730, 240.492] = 3.513, *p* = 0.038, partial η^2^ = 0.025). Post-hoc comparisons revealed no significant differences of VAS_F scores in DRL and BL immediately before the intervention (DRL, 52.5 ± 24.7; BL. 51.5 ± 25.3, *p* > 0.05), but significantly lower VAS_F scores immediately after the intervention (DRL, 43.1 ± 22.8, BL, 38.4 ± 19.5, *p* = 0.023) and at 13:00 (DRL: 46.8 ± 22.3, BL, 40.8 ± 19.4, *p* = 0.005) in BL compared to DRL. Additionally, we observed a significant main effect for the factor intervention (F[1, 139] = 5.384, *p* = 0.022, partial η^2^ = 0.037), with significantly lower overall mean VAS_F scores with BL (overall: 42.8 ± 22.2) than with DRL (overall: 47.4 ± 23.2, *p* = 0.022; Fig. 4). Further, we found a significant main effect for measurement time (F[1.535, 213.383] = 26.804, *p* < 0.001, partial η^2^ = 0.162). Post-hoc comparisons revealed significantly lower VAS_F scores immediately after the intervention (39.8 ± 21.3, *p* < 0.001) and at 13:00 (44.4 ± 21.1, *p* < 0.001) than immediately before the intervention (51.1 ± 25.0), but no significant difference was found between measurements immediately after the intervention and at 13:00 (*p* > 0.05).

#### Quality of life throughout the intervention weeks

MQLI scores were included in a repeated measures ANOVA with the factors of intervention (DRL, BL) and measurement time (baseline, day 2, day 4, day 6, day 7), which yielded no significant main effects or interaction effect (*p* > 0.05). MQLI scores over the course of the week are depicted in Fig. 3b.

### Feasibility

#### Side effects

The number of daily reported side effects and their severity rated on a 6-point Likert scale from 0 (not at all) to 5 (very strong) were low with no or hardly any notice of side effects in around 85.6% of ratings after BL and 89.1% of ratings after DRL. Glare was the most frequently reported side effect, with one report of strong glare and one report of very strong glare using DRL as well as six reports of strong glare using BL. No other strong or very strong side effects were reported. Detailed statistical analyses of reported side effects per intervention day comparing DRL and BL, along with a distribution of side effects severity, can be found in the Supplementary Materials, Appendix C. One participant discontinued study participation during the first week, after 3 days of DRL, due to severe glare. No other participant reported severe side effects that led to discontinuation of trials.

#### Sleep parameters

Comparing subjective sleep quality data, two-factor ANOVAs with repeated measures with the factors of measurement day (baseline to day 7 [follow-up]) and intervention (DRL, BL) were calculated for each parameter. A significant main effect of measurement day regarding sleep quality was found (F[7, 133] = 2.466, *p* = 0.021, partial η^2^ = 0.115), with a significantly lower sleep quality reported on day 3 (Monday, 62.9 ± 19.4) compared to day 2 (Sunday, 73.3 ± 17.7, *p* = 0.043), but no main effect of intervention or interaction effect were observed. The remaining sleep parameters (sleep latency, time in bed, total sleep time, and sleep efficiency) yielded no significant main effects nor interaction effect (all *p* > 0.05). Finally, the Student’s *t*-test for dependent measures revealed significantly more naps on intervention days during the BL week (2.1 ± 2.3) than in the DRL week (1.3 ± 1.4; *t* = − 2.223, *p* = 0.039, *n* = 20, Cohen’s *d* = 0.45).

#### Comfort ratings

Compared to the DRL intervention, participants rated the BL glasses significantly more positively regarding their ability to increase fitness (Cohen’s *d* = 0.64) and well-being (Cohen’s *d* = 0.64), have a positive effect on the morning (Cohen’s *d* = 0.52), and to reduce fatigue (Cohen’s *d* = 0.66), but also to negatively affect patients’ view due to the brightness (Cohen’s *d* = 0.51; Table 5). The BL intervention was also rated to be more fitness-increasing (Cohen’s *d* = 0.66) and more activating (Cohen’s *d* = 0.61) than DRL. Regarding the willingness to recommend the intervention and ratings using a “school grading” system both were higher for the BL than for the DRL intervention, but these differences were not significant.

**Table 5:** Comfort ratings for the dim red and blue light glasses.

	Descriptive statistics				Inferential statistics	
	DRL intervention		BL intervention			
	*Mdn*	*M (SD)*	*Mdn*	*M (SD)*	*z*	*p*
Rating for intervention (5-point Likert scale: 1 = not at all; 5 = very): The light glasses…						
…increased fitness	2	2.2 (1.1)	3	2.9 (1.1)	− 2.636	.008
…increased well-being	3	2.5 (1.2)	4	3.6 (1.0)	− 2.623	.009
…facilitated waking up	3	3.1 (1.2)	4	3.9 (1.2)	− 1.951	.051
…facilitated wakefulness	3	2.7 (1.3)	3	3.5 (1.1)	− 1.812	.070
…had a positive effect on the morning	3	3.0 (1.3)	4	4.0 (1.0)	− 2.129	.033
… positively influenced concentration	3	2.7 (1.1)	3	3.3 (0.9)	− 1.632	.103
…were irritating	1	1.5 (0.8)	1	1.3 (0.9)	0.000	> .999
…irritated the eyes	1	1.7 (0.9)	1	1.8 (1.0)	− 0.577	.564
…brightness negatively affected the view	2	1.8 (0.9)	3	2.7 (1.1)	− 2.095	.036
…disturbed reading	2	1.8 (0.8)	2	2.5 (1.0)	− 1.930	.054
…generated disturbing reflections in the smartphone screen	2	1.9 (0.8)	2	2.0 (0.8)	− 1.000	.317
…reduced fatigue	3	2.4 (1.0)	3	3.5 (0.9)	− 2.658	.008
Semantic differential (7-point Likert scale: 1 = very…; 4 = neither nor; 7 = very…)						
pleasant—unpleasant	4	3.5 (1.2)	3	2.9 (1.3)	− 1.292	.196
fitness increasing—fitness decreasing	4	3.9 (1.3)	3	2.6 (1.1)	− 2.641	.008
drowsing—activating	4.5	4.6 (1.4)	6	5.8 (1.1)	− 2.448	.014
not disturbing—disturbing	3	2.9 (1.4)	2	2.7 (1.5)	− 0.177	.859
too short—too long	4	3.7 (1.4)	4	3.4 (1.1)	− 0.568	.570
weak—strong	4	4.3 (1.3)	4	4.6 (1.2)	− 0.480	.631
Overall rating for intervention (6-point Likert scale: 1 = not at all; 6 = absolutely)						
Recommendation	4	4.2 (1.6)	5	4.9 (1.0)	− 1.467	.143
Evaluation of the intervention (6-point Likert scale: 1 = very good; 6 = insufficient)						
Grade (i.e., school grade)	3	3.1 (1.4)	2	2.4 (0.9)	− 0.975	.330

DRL, dim red light; BL, blue-enriched light; *Mdn*, median; *M*, mean; *SD*, standard deviation.

Inferential statistics were calculated using the Wilcoxon signed-rank test comparing ratings for the DRL (dim red light) and BL (blue light) intervention

## Discussion

In this exploratory field study, we evaluated the efficacy and feasibility of a novel light therapy method (blue-enriched light vs. dim red light exposure immediately after waking up) using light therapy glasses to treat MS-related fatigue.

Our main findings indicated good efficacy of both light glasses: 1 week of both light interventions significantly reduced fatigue levels assessed using the FSS total score. Reductions in FSS fatigue levels with BL indicated clinically significant changes, which were not observed with DRL. Further, we found acute effects of the light interventions on VAS_F-measured fatigue levels immediately after exposure and hours later (at 13:00), with significantly higher effects with BL than with DRL. The feasibility of our light treatment was high, and both light interventions were well tolerated. Patients reported a higher severity of side effects regarding overexertion (on two days), stinging eyes (on one day), and glare (on four days) using BL, with significantly better ratings for these glasses than for DRL regarding items such as increasing fitness and well-being, positively influencing the morning, and, importantly, reducing fatigue. Subjectively assessed sleep parameters revealed no negative effects of the light interventions on sleep, but patients reported taking significantly more naps during the day when using the BL than with the DRL glasses.

A strength of the present study is the temporal resolution at which fatigue was assessed using the VAS_F. This allowed explorations of the effects of both interventions on the temporal dynamics of MS-related fatigue immediately after light treatment and hours later. Thus, this study provides first evidence for the slight superiority of BL, compared to DRL, in reducing fatigue levels immediately after light exposure as well as for a prolonged duration after light exposure. Considering the small sample size included in the present study, these findings should be interpreted with caution and need to be validated using bigger sample sizes. Comparing baseline with follow-up values, our results add to the findings of past research^42,43^ by demonstrating a reduction of MS-related fatigue levels in both interventions after only 1 week of usage. Further, we also found clinically significant changes in FSS scores, but only with BL. In contrast to past research, we further analysed each participants’ individual changes in FSS scores throughout the intervention weeks and found variations between patients. Some patients responded only to BL, some responded only to DRL, some responded to both interventions, and some did not respond at all. This highlights stark inter-individual variability of the effects of light therapy and poses the question of whether there are responders and non-responders to light therapy among patients MS and fatigue, as observed in studies evaluating light therapy for SAD^60^. This hypothesis should be followed up on in further research, especially with bigger sample sizes, since fatigue is a highly subjective symptom influenced by many factors (daily activities and sleep, mood, and daily light exposure) which might also explain some variability^61–63^. Further, it highlights the need for examining individualized intervention methods for MS-related fatigue.

Another strength of our study is the daily assessment of side effects immediately after the light exposure, which also allowed a high temporal resolution in measurements of the severity of possible side effects as well as their development over the course of the intervention weeks. The reported side effects and their severity in our study were mild, and more severe side effects were observed early during the intervention weeks, potentially indicating that patients took a short time to adjust to the light exposure using glasses, as postulated in other research^64^. The differences in side effects and their severity observed between BL and DRL may in part be explained by the tenfold higher corneal illuminance in the BL intervention than in the DRL intervention. Further, the observed side effects and their severity in this study are negligible in comparison to those adverse effects experienced using pharmacological interventions for MS-related fatigue^18,19^. Along with the very low number of treatment discontinuations (1 patient during DRL) and the demonstrated reductions in fatigue levels, our results speak in favour of using light therapy to treat MS-related fatigue.

Within the framework of the study protocol, we focused on enabling patients to implement trial participation into their everyday lives. Interestingly, adherence rates were high at 96%, and only one person discontinued participation due to severe side effects with DRL intervention.

Patients reported significantly more naps during the BL intervention week than the DRL week. This raises the question if the light glasses’ positive effects on fatigue levels during the morning led to increased sleepiness after 13:00. It may also put into perspective that the D-FIS results (see supplementary materials) did not significantly change from baseline to follow-up: the D-FIS was administered in the evening and may have been affected by increased sleepiness following BL and, as reported in past research^65^, the possibility of an earlier melatonin onset following BL exposure. Since proper conduction and storage of melatonin samples is difficult in field studies such as this, a follow-up study using a laboratory study design could investigate this aspect.

In general, there are stark differences in how different measurements of fatigue operationalise fatigue, based on different definitions of this debilitating symptom^1^. This offers another explanation on variations in fatigue results across different questionnaires, such as the VAS_F, D-FIS, and FSS in our study. Further, there is a history of strong placebo effects in the pharmacological^18^ and non-pharmacological treatment^66^ of MS-related fatigue. Patients with MS often experience a strong placebo response, which would require a very large intervention effect to surpass. Studies on light interventions to treat MS-related fatigue are scarce, with our study being the third to have examined this method for MS-related fatigue. A review on light therapy for neurodegenerative disorders demonstrated that this treatment option offers attractive approaches for these types of diseases^67^, highlighting the need for further research on the topic to refine light therapies for treating MS-related fatigue.

Our study comes with several limitations. Despite screening 70 interested patients, only 20 were included in the final analyses due to the exclusion criteria. This limits the power of our analyses and restricts the generalizability of our finding, especially considering we conducted a randomised controlled trial and aimed at achieving a sample size with a minimum of n = 25. Future research should include a larger sample size and more advanced disease severity, which might produce more robust findings. The DRL condition utilized in our study was intended to serve as a control condition. However, dim red light might be unsuitable as control, since research has shown that dim red light also influences alertness and attention during day- and nighttime^68,69^ and has an impact on mood^39^. The challenges of selecting and implementing a valid control condition in light intervention studies has been addressed in recent literature^26^. Future research may help to disentangle the effects of participant expectations from the potential effects of dim and bright light administration by including a control condition with an alleged non-visual light as implemented by Popp et al.^70^ to provide evidence for the robustness of our findings. Further, many of our included patients showed suspicious values regarding sleep disorders during screening. Past research has implemented a more vigorous exclusion of these patients as well as specific chronotypes^43^ and demonstrated that treating sleep disorders can significantly improve MS-related fatigue^71^. The occurrence and underdiagnosis of sleep disorders is, however, relatively common in patients with MS^72–74^, which is reflected in the screening results of our sample, since no patients reported pre-diagnosed sleep disorders. According to the manufacturer of the light therapy glasses, the effects of the light glasses should be noticeable within 4 to 6 days of usage^75^. Therefore, as a proof of principle, we chose an intervention period of 7 days per intervention, since our study protocol was already rather intense, with 119 questionnaires required over the course of 22 days. For a symptom as severe as MS-related fatigue, regular usage over a longer period might be necessary to evoke more consistent and profound reductions in fatigue levels than we found and give insights into sustained long-term effects, adaptation, and loss of efficacy. These issues should be considered in future research including longer observation periods and follow-up assessments. Studies debate seasonal changes in MS-related fatigue in relation to the outdoor temperature, with increased fatigue severity linked to warmer months^76^, but findings remain inconclusive^77^. Further, increased exposure to sunlight was reportedly linked to reduced fatigue levels^63^. In our study, we did not monitor daily light exposure or temperature levels. However, our enrolment phase spanned from December 2023 to July 2025, and we included 12 patients who participated during winter and early spring (standard time) and 8 patients who participated during late spring and summer (daylight saving time), covering the majority of seasons. Further research should consider monitoring outdoor temperature and illuminance levels to control for their effects on MS-related fatigue during light exposure trials.

## Conclusions

The present exploratory field study examined a novel light therapy method for MS-related fatigue, providing evidence for immediate beneficial effects of light therapy on MS-related fatigue, both over the course of the intervention week (FSS, VAS_F) and within hours of usage (VAS_F). Further, the intervention using blue-enriched light (BL) showed stronger effects to dim red light (DRL) in reducing fatigue immediately after usage as well as for a prolonged time. Our small sample size and low power, the lack of a biologically inert placebo, and the relatively short observation period of 1 week per intervention limited the generalizability of our results. Therefore, further research should include larger, less restricted samples of fatigued people with MS and fatigue for a longer duration and implementing alternative control conditions. Importantly, using light-emitting glasses after waking up in the morning was well tolerated with high feasibility, providing initial evidence for a promising intervention method that could help patients with MS-related fatigue.

## Supplementary Information

Supplementary Information

## Data Availability

Study data are available from the corresponding author on reasonable request.
