# Supplementary material for: Exploratory crossover field study indicates efficacy and feasibility of light therapy glasses to mitigate fatigue in multiple sclerosis
Source: Sci Rep. 2026 Jun 23;16:19533. doi: 10.1038/s41598-026-56950-3 (PMC13291330; doi:10.1038/s41598-026-56950-3)
Supplement: Supplementary file 1 — Supplementary Information [file 41598_2026_56950_MOESM1_ESM.docx]

Exploratory crossover field study indicates feasibility and efficacy of light therapy glasses to counteract fatigue in multiple sclerosis

Supplementary Materials

Julia Ottersbach^1,2^, Markus Canazei^3^, Sophie K. Bauer^1,2^, Celine Hfalek^1,2^, Caren Berggold^1,2^, Thomas C. Wetter^1^, De-Hyung Lee^4^, Ralf A. Linker^4^, Roland F. J. Popp^1^

1 Department of Psychiatry and Psychotherapy, Center of Sleep Medicine, University of Regensburg, 93053 Regensburg, Germany

2 Department of Experimental Psychology, University of Regensburg, 93053 Regensburg, Germany

3 Department of Psychology, University of Innsbruck, 6020 Innsbruck, Austria

4 Department of Neurology, University of Regensburg, 93053 Regensburg

Appendix A: Detailed description of screening and outcome measures

*Screening for sleep disorders*

- The **Berlin Questionnaire for sleep apnoea [**^1^] is a self-administered 10-item questionnaire regarding three categories related to sleep apnoea (snoring, daytime sleepiness, and obesity or hypertension). The outcomes define a high risk for obstructive sleep apnoea if a patient shows risk factors in at least two of the three categories. The Berlin Questionnaire shows sufficient internal consistency (Cronbach’s α varies between .68 and .98) and test–retest reliability (Cohen’s κ varies between .74 and .98).
- Screening for **restless legs syndrome** (RLS) was based on the five essential diagnostic criteria defined by the International RLS Study Group [^2^].
- The **Regensburg Insomnia Scale** (RIS) [^3^] is a 10-item self-administered questionnaire assessing cognitive, emotional, and behavioural aspects of psychophysiological insomnia. Participants respond by rating questions on a scale from 0 (*always*) to 4 (*never*), which are summed to a total score of 0–40, with higher scores indicating higher levels of insomnia. Total scores >12 indicate symptoms of psychophysiological insomnia ^3^. The internal consistency of the RIS is good, with a Cronbach’s α of .89.

*Screening for daytime sleepiness*

- The **Epworth Sleepiness Scale** (ESS) [^4^] is a self-administered questionnaire on subjective daytime sleepiness. The ESS contains eight questions about the probability of falling asleep or dozing off (i.e., subjective sleep propensity) while engaged in different daily activities. A global score is derived from adding the responses (each rated from 0–3; the global score ranges from 0–24). The ESS shows sufficient internal consistency (Cronbach’s α = .88) and test–retest reliability (r = .82) [^5^]. Participants in the present study were administered the German version of the ESS [^6^]. According to a German validation study, scores higher than 10 categorize a participant’s daytime sleepiness as ‘clinically suspicious’, while scores > 12 define it as ‘clinically relevant’ [^6^].

*Fatigue*

The following three questionnaires were used to assess fatigue levels during screening and intervention weeks.

- Using a **Visual Analogue Scale for Fatigue** (VAS_F), participants rated their current fatigue levels from 0% (no fatigue) to 100 (very strong fatigue), serving as primary outcome measure.
- The **Fatigue Severity Scale** (FSS) [^7^] is a nine-item questionnaire that assesses the severity of fatigue during the week prior to testing on a 7-point scale from 1 (*disagree*) to 7 (*agree*). The total score, used as primary outcome measure in the current study, ranges from 9 to 63, with values >36 indicating fatigue [^8–10^]. The internal consistency of the FSS is high, with a Cronbach’s α of .94. The German version of the FSS was used in the current study with test–retest reliability of the individual items ranging from *r* = .63 to *r* = .88 [^11^].
- The **Daily Fatigue Impact Scale** (D-FIS) [^12^], an eight-item questionnaire based on the Fatigue Impact Scale ^13^, was used every other evening during participation. Respondents rate the effects of fatigue on daily life on a five-point Likert scale from 0 (*no problem*) to 4 (*extreme problem*). The sum score ranges from 0 to 32, with higher scores indicating stronger effects of fatigue. For the current study, a non-validated German version of the D-FIS was created by the authors by means of forward-and-back translation.

*Screening for Sleep Quality*

- The **Pittsburgh Sleep Quality Index** (PSQI) [^14^] is a self-administered questionnaire that assesses sleep quality and disturbances over a 1-month period. The PSQI contains 19 items from which seven sleep component scores are derived (e.g., subjective sleep quality, sleep latency). A global sleep quality score can be calculated as the sum of the component scores with a minimum of 0 and a maximum of 21. This score can be used to group participants as having generally good (score of 5 or lower) or bad sleep (score above 5). The PSQI shows sufficient internal consistency with a Cronbach’s α of .83, a sensitivity of 89.6%, a specificity of 86.5%, and sufficient test–retest reliability of *r*=.88 [^14^]. In the present study, a validated German version of the PSQI was used during screening [^15^].

*Screening for Chronotype*

- The **Morningness–Eveningness Questionnaire (MEQ)** assesses the chronotype. Responses to its 19 questions regarding performance, sleep behaviour, and well-being within a 24-hour time frame are summed to provide a total score with a maximum of 86. This score indicates one of five chronotypes: definite evening (14–30), moderate evening (31–41), neutral (42–58), moderate morning (59–69), and definite morning type (70–86). In the present study, the German version of the MEQ, called the D-MEQ, was used during screening. It shows high internal consistency with Cronbach’s α = .82 [^16^].

*Mental health, quality of life*

- The **Beck Depression Inventory-II** (BDI-II) [^17^] is a 21-item questionnaire assessing the severity of depression symptoms such as sadness, self-harm, and loss of appetite in the two weeks prior to testing on a four-point scale from 0–3. The total score ranges from 0–63 indicating mild (14–19), moderate (20–28), and severe depression (29–63) [^18,19^]. The BDI-II shows high internal consistency (Cronbach’s α of .91) [^17^] and good to excellent test–retest reliability (*r* = .73–.96) [^20^]. Participants were administered the German version of the BDI-II in the current study during screening ^21^.
- The **Multicultural Quality of Life Index** [^22^] assesses quality of life in 10 dimensions using 10 questions regarding physical well-being, psychological/emotional well-being, self-care and independent functioning, occupational functioning, interpersonal functioning, social emotional support, community and services support, personal fulfilment, spiritual fulfilment, and overall quality of life. Participants rate each domain from 1 (*poor*) to 10 (*excellent*). The total score consists of the summated scores from all answered items and ranges from 10 to 100. The internal consistency (Cronbach’s α) ranges from .90 to .92, its test–retest reliability was reported at *r* = .87 [^22^]. The German version of the Multicultural Quality of Life Index was used in the current study on every second evening during intervention weeks [^23^].

*Side effects*

Assessing side effects from using the light glasses’, a questionnaire on **Asthenopic Complaints** was used to query and rate the following side effects on a 6-point scale (0 [*not at all noticeable*]; 1 [*hardly noticeable*]; 2 [*a little noticeable*] 3 [*noticeable*]; 4 [*strong*]; 5 [*very strong*]): *Overexertion of the eyes, headache, watery eyes, stinging eyes, burning eyes, blurred vision, pain in and around the eyes, glare, dizziness*. The questionnaire used selected items of a long version of the questionnaire reported in Leichtfried et al. [^24^] based on an article by Terman and Terman [^25^]. Side effects were assessed after usage of the light glasses on every intervention day.

*Sleep*

A **sleep diary** was used to assess patients sleep throughout study participation. From patients’ responses, sleep quality (%), total sleep time (min; calculated as the difference between reported time falling asleep and reported time waking up), time in bed (min; calculated as the difference between reported bed time and reported time waking up), sleep latency (min; calculated as the difference between reported bed time and reported time falling asleep), and sleep efficiency (%; calculated as the ratio between reported total sleep time and time in bed) were extracted for further analyses.

*Comfort ratings*

**Comfort ratings** were used to assess and compare participants’ acceptance of the interventions. Beneficial and visual side effects of the light intervention were evaluated on a five-point scale (1 [*fully disagree*]; 2 [*rather disagree*]; 3 [*partly agree*]; 4 [*rather agree*]; 5 [*fully agree*]). The beneficial effects questions included the items ‘The light glasses increased my fitness’, ‘The light glasses increased my well-being’, ‘The light glasses facilitated wakefulness’, ‘The light glasses had a positive effect on the morning’, and ‘The light glasses positively influenced my concentration’. The side effects questionnaire comprised six items: ‘The light glasses were irritating’, ‘The light glasses disturbed me’, ‘The light glasses irritated my eyes’, ‘The light glasses negatively affected my view’, ‘The light glasses disturbed reading’, and ‘The light glasses generated disturbing reflections in the smartphone screen’. We used a seven-point semantic differential ranging from 1 (*very…*) via 4 (*neither nor*) to 7 (*very…*) with the following items: ‘pleasant – unpleasant’, ‘fitness increasing - fitness decreasing’, ‘somniferous – activating’, ‘not disturbing – disturbing’, ‘too short – too long’, ‘in the foreground – in the background’, and ‘weak – strong’. Participants stated whether they would recommend the light glasses to other night shift workers on a scale from 1 (*not at all*) to 6 (*absolutely*) and gave the light glasses school grades from 1 (*very good*) to 6 (*insufficient*).

Appendix B: Outlier and missing data replacement

One outlier (FSS score = 9) was identified and replaced by the mean of that person’s previous and subsequent data in the same intervention week. If baseline values were missing from one intervention week, these were replaced with that same person’s baseline values from the other intervention week. Missing values during the test weeks were filled in with the mean value of that person’s data recorded one measurement day before and after within the same intervention week (VAS_F, 58 missing values out of 960 values were substituted [7%]; FSS, 15 missing values out of 200 values were substituted [8%]; D-FIS, 8 missing values out of 260 values were substituted [2%]; MQLI, 8 missing values out of 200 values were substituted [4%]; Sleep protocol, 100 missing values out of 2.020 values were substituted [5%]; side effects, 144 missing values out of 2.520 values were substituted [6%]). Comfort questionnaires were administered once at the end of each intervention period. We had missing data from two patients in the dim red light intervention and one in the blue-enriched light intervention. To run matched pairs analyses, data from 17 persons for whom comfort ratings were available for both interventions were included in the analyses.

Appendix C: Additional results

Comparison of baseline values at the beginning of the BL and DRL intervention weeks

**Table S1** Baseline scores at the beginning of both intervention weeks

|  | DRL | BL |  |  |
| --- | --- | --- | --- | --- |
|  | *M ± SD* | *M ± SD* | *t(19)* | *p* |
| VAS_F pre | 51.3 ± 24.4 | 51.0 ± 25.5 | 0.036 | .972 |
| VAS_F post | 43.3 ± 22.8 | 48.8 ± 21.5 | -1.255 | .225 |
| VAS_F at 13:00 | 51.0 ± 26.1 | 59.0 ± 22.0 | -1.152 | .264 |
| FSS | 48.8 ± 8.1 | 50.1 ± 8.0 | -1.136 | .270 |
| MQLI | 60.4 ± 17.2 | 65.2 ± 9.4 | -1.349 | .193 |

**Note**: DRL, dim red light intervention; BL, blue-enriched light intervention; *M*, mean, *SD*, standard deviation, VAS_F, Visual Analogue Scale for Fatigue; pre: before the intervention after waking up; post: after the intervention; FSS, Fatigue Severity Scale, MQLI, Multicultural Quality of Life Index

Inferential statistics were calculated using Student’s *t*-test for dependent measures

Descriptive and inferential results of the Daily Fatigue Impact Scale

*Comparison of baseline values at the beginning of the BL and DRL intervention weeks*

Patients’ scores on the D-FIS assessed at baseline of the BL (18.9 ± 8.4) and DRL (18.4 ± 5.4) intervention weeks did not differ significantly (t[19] = –0.396, p = 0.697) between conditions.

*Comparison of baseline and follow-up scores*

Comparing D-FIS scores between baseline and follow-up, a reduction of 2.8 points was seen after 1 week of DRL (baseline: 18.4 ± 5.4; follow-up: 15.6 ± 6.6), and a reduction of 2.9 points after 1 week of BL (baseline: 18.9 ± 8.4, follow-up: 16.0 ± 4.9). In a two-factor repeated measures ANOVA, no significant main effects or interaction effect were seen (all *p* > .05).

*D-FIS-measured fatigue throughout the intervention weeks*

D-FIS scores assessed throughout the intervention weeks were compared in a repeated measures ANOVA with the factors of intervention (DRL, BL) and measurement day (baseline, day 2, day 4, day 6, day 7). No main effects or interaction effect were found (*p* > 0.05).

Vision-related side effects of the interventions

When examining reported side effects per intervention day between DRL and BL, Wilcoxon signed-rank test revealed significantly more severe overexertion on day 1 (Saturday; DRL, 0.7 ± 1.0; BL, 1.2 ± 1.1, *z* = –2.301, *p* = 0.021, Cohen’s *d* = 0.51) and day 2 (Sunday; DRL, 0.6 ± 0.8; BL, 1.0 ± 1.0, *z* = –2.138, *p* = 0.033, Cohen’s *d* = 0.48), significantly more severe stinging eyes on day 3 (Monday; DRL, 0.3 ± 0.6; BL, 0.5 ± 0.7; *z* = –2.000, *p* = 0.046, Cohen’s *d* = 0.44), and significantly more severe glare on day 1 (Saturday, DRL, 0.7 ± 1.2; BL, 1.7 ± 1.4; *z* = –2.293; *p* = 0.022, Cohen’s *d* = 0.51), day 4 (Tuesday; DRL, 0.6 ± 0.9; BL, 1.4 ± 1.1; *z* = –2.801, *p* = 0.005, Cohen’s *d* = 0.63), day 5 (Wednesday; DRL, 0.7 ± 0.9; BL, 1.4 ± 1.2; *z* = –2.041, *p* = 0.041, Cohen’s *d* = 0.46), and day 6 (Thursday; DRL, 0.9 ± 1.0; BL, 1.5 ± 1.1; *z* = –2.209, *p* = 0.027, Cohen’s *d* = 0.49) when using BL than with DRL. No differences were found on any other intervention day regarding these side effects, nor on any intervention day at all regarding headache, watering eyes, itchy eyes, blurred vision, pain in and around the eyes, and dizziness (*p* > 0.05). Table S2 shows the results of the Wilcoxon signed-rank test for all items and intervention days, and Figure S1 depicts the distribution of side effects severity in both study interventions.

**Table S2** Results from the Wilcoxon signed-rank test comparing side effects between the DRL and BL condition for each intervention day

|  | **Day 1** |  | **Day 2** |  | **Day 3** |  | **Day 4** |  | **Day 5** |  | **Day 6** |  | **Day 7** |  |
| --- | --- | --- | --- | --- | --- | --- | --- | --- | --- | --- | --- | --- | --- | --- |
|  | *z* | *p* | *z* | *p* | *z* | *p* | *z* | *p* | *z* | *p* | *z* | *p* | *z* | *p* |
| Overexcertion of the eyes | -2.301 | **.021** | -2.138 | **.033** | -1.897 | .058 | -0.378 | .705 | -1.081 | .279 | -0.586 | .558 | -0.725 | .468 |
| Headache | -0.526 | .599 | -1.414 | .157 | -0.368 | .713 | -0.707 | .480 | -0.756 | .450 | -0.587 | .557 | 0.000 | >.999 |
| Watery eyes | -1.328 | .167 | -0.378 | .705 | -0.090 | .928 | -1.324 | .180 | -0.333 | .739 | -0.712 | .476 | -0.137 | .891 |
| Itchy eyes | -1.414 | .157 | 0.000 | >.999 | -1.236 | .216 | 0.000 | >.999 | -1.414 | .157 | -0.730 | .465 | -0.707 | .480 |
| Stinging eyes | -0.756 | .450 | -1.134 | .257 | -2.000 | **.046** | -0.216 | .829 | -1.236 | .216 | -0.121 | .904 | -2.000 | **.046** |
| Blurred vision | -1.754 | .079 | -0.061 | .951 | -1.754 | .079 | -1.300 | .194 | -0.750 | .453 | -1.035 | .301 | 0.000 | >.999 |
| Pain in/around the eyes | -0.816 | .414 | -1.000 | .317 | -0.707 | .480 | -0.557 | .577 | 0.000 | >.999 | -1.289 | .197 | 0.000 | >.999 |
| Glare | -2.293 | **.022** | -1.567 | .117 | -1.917 | .055 | -2.801 | **.005** | -2.041 | **.041** | -2.209 | **.027** | -0.956 | .339 |
| Dizziness | -1.414 | .157 | 0.000 | >.999 | -1.633 | .102 | -1.000 | .317 | 0.000 | >.999 | -0.447 | .655 | -1.000 | .317 |

**Note:** DRL, dim red light; BL, blue-enriched light; Day 1, Saturday; Day 2, Sunday; Day 3, Monday; Day 4, Tuesday; Day 5, Wednesday; Day 6, Thursday; Day 7, Friday, follow-up.

Printed in bold are *p*-Values < 0.05.

**Fig. S1.** Side effects during the intervention sessions.

**(b)**

**(a)**

**Note:** Reported side effects severity during **(a) dim red light intervention** sessions and **(b) blue-enriched light intervention** sessions with ratings ‘not at all’ (green), ‘hardly’ (yellow), ‘a little’ (light orange), ‘noticeable’ (dark orange), ‘strong’ (bright red), and ‘very strong’ (dark red) noticeable.

References

1. Netzer, N. C., Stoohs, R. A., Netzer, C. M., Clark, K. & Strohl, K. P. Using the Berlin Questionnaire To Identify Patients at Risk for the Sleep Apnea Syndrome. *Ann. Intern. Med.* **131**, 485–491 (1999).

2. Allen, R. P. *et al.* Restless legs syndrome/Willis-Ekbom disease diagnostic criteria: updated International Restless Legs Syndrome Study Group (IRLSSG) consensus criteria--history, rationale, description, and significance. *Sleep Med.* **15**, 860–873 (2014).

3. Crönlein, T. *et al.* Regensburg Insomnia Scale (RIS): a new short rating scale for the assessment of psychological symptoms and sleep in insomnia; Study design: development and validation of a new short self-rating scale in a sample of 218 patients suffering from insomnia and 94 healthy controls. *Health Qual. Life Outcomes* **11**, 65 (2013).

4. Johns, M. W. A New Method for Measuring Daytime Sleepiness: The Epworth Sleepiness Scale. *Sleep* **14**, 540–545 (1991).

5. Johns, M. W. Reliability and Factor Analysis of the Epworth Sleepiness Scale. *Sleep* **15**, 376–381 (1992).

6. Sauter, C. *et al.* Normative Values of the German Epworth Sleepiness Scale. *Somnologie - Schlafforschung Schlafmed.* **11**, 272–278 (2007).

7. Krupp, L. B., LaRocca, N. G., Muir-Nash, J. & Steinberg, A. D. The Fatigue Severity Scale: Application to Patients With Multiple Sclerosis and Systemic Lupus Erythematosus. *Arch. Neurol.* **46**, 1121–1123 (1989).

8. Andreasen, A., Stenager, E. & Dalgas, U. The effect of exercise therapy on fatigue in multiple sclerosis. *Mult. Scler. J.* **17**, 1041–1054 (2011).

9. Learmonth, Y. C. *et al.* Psychometric properties of the Fatigue Severity Scale and the Modified Fatigue Impact Scale. *J. Neurol. Sci.* **331**, 102–107 (2013).

10. Lerdal, A., Wahl, A., Rustøen, T., Hanestad, B. R. & Moum, T. Fatigue in the general population: a translation and test of the psychometric properties of the Norwegian version of the fatigue severity scale. *Scand. J. Public Health* **33**, 123–130 (2005).

11. Reske, D., Pukrop, R., Scheinig, K., Haupt, W. F. & Petereit, H.-F. Messbarkeit von Fatigue bei multipler Sklerose mithilfe standardisierter Methoden im deutschsprachigen Raum. *Fortschritte Neurol. · Psychiatr.* **74**, 497–502 (2006).

12. Fisk, J. D. & Doble, S. E. Construction and validation of a fatigue impact scale for daily administration (D-FIS). *Qual. Life Res. Int. J. Qual. Life Asp. Treat. Care Rehabil.* **11**, 263–272 (2002).

13. Fisk, J. D. *et al.* Measuring the functional impact of fatigue: initial validation of the fatigue impact scale. *Clin. Infect. Dis. Off. Publ. Infect. Dis. Soc. Am.* **18 Suppl 1**, S79-83 (1994).

14. Buysse, D. J., Reynolds III., C. F., Monk, T. H., Berman, S. R. & Kupfer, D. J. The Pittsburgh Sleep Quality Index: a new instrument for psychiatric practice and research. *Psychiatry Res.* **28**, 193–213 (1989).

15. Backhaus, J., Junghanns, K., Broocks, A., Riemann, D. & Hohagen, F. Test–retest reliability and validity of the Pittsburgh Sleep Quality Index in primary insomnia. *J. Psychosom. Res.* **53**, 737–740 (2002).

16. Griefahn, B., Künemund, C., Bröde, P. & Mehnert, P. Zur Validität der deutschen Übersetzung des Morningness-Eveningness-Questionnaires von Horne und Östberg. *Somnologie* **5**, 71–80 (2001).

17. Beck, A. T., Steer, R. A., Ball, R. & Ranieri, W. Comparison of Beck Depression Inventories -IA and -II in psychiatric outpatients. *J. Pers. Assess.* **67**, 588–597 (1996).

18. Beck, A. T., Steer, R. A. & Brown, G. Beck Depression Inventory–II. (1996) doi:10.1037/t00742-000.

19. Smarr, K. L. & Keefer, A. L. Measures of depression and depressive symptoms: Beck Depression Inventory-II (BDI-II), Center for Epidemiologic Studies Depression Scale (CES-D), Geriatric Depression Scale (GDS), Hospital Anxiety and Depression Scale (HADS), and Patient Health Questionnaire-9 (PHQ-9). *Arthritis Care Res.* **63**, S454–S466 (2011).

20. Wang, Y.-P. & Gorenstein, C. Psychometric properties of the Beck Depression Inventory-II: a comprehensive review. *Braz. J. Psychiatry* **35**, 416–431 (2013).

21. Hautzinger, M., Keller, F. & Kühner, C. *Beck Depressions-Inventar: BDI II.Revision*. (Harcourt Test Services, Frankfurt am Main, 2006).

22. Mezzich, J. E., Cohen, N. L., Ruiperez, M. A., Banzato, C. E. M. & Zapata-Vega, M. I. The Multicultural Quality of Life Index: presentation and validation. *J. Eval. Clin. Pract.* **17**, 357–364 (2011).

23. Saletu, B. *et al.* Quality of life in nonorganic and organic sleep disorders: I. Comparison with normative data. *Wien. Klin. Wochenschr.* **115**, 246–254 (2003).

24. Leichtfried, V. *et al.* Bright light therapy: Minimizing light induced side effects with an innovative light setup. *Int. J. Psychiatry Clin. Pract.* **14**, 309–312 (2010).

25. Terman, M. & Terman, J. S. Bright light therapy: side effects and benefits across the symptom spectrum. *J. Clin. Psychiatry* **60**, 799–808; quiz 809 (1999).
